# Healthcare Professionals’ Perceptions of the Palliative Care Needs of Patients with Severe Brain Injury and Their Caregivers: A Qualitative Study

**DOI:** 10.3390/brainsci16050482

**Published:** 2026-04-30

**Authors:** Flavia Primosa, Serena Cazzato, Lucia Gotri, Romano Marchini, Orejeta Diamanti, Laura Iacorossi, Andreina Saba

**Affiliations:** 1 Intercompany Department of Health Professions, AUSLPR, Department of Medicine and Surgery, University of Parma, 43121 Parma, Italy; flavia.primosa@unipr.it (F.P.); serena.cazzato@unipr.it (S.C.); lucia.gotri@unipr.it (L.G.); 2 Intercompany Department of Health Professions, Local Health Authority of Parma (AUSLPR), 43121 Parma, Italy; rmarchini@ausl.pr.it; 3 Scientific Direction, Veneto Institute of Oncology (IOV), Scientific Institute for Research, Hospitalization and Healthcare (IRCCS), 35128 Padua, Italy; orejeta.diamanti@iov.veneto.it; 4 Department of Life, Health Sciences and Health Professions, Link Campus University, 00165 Rome, Italy; 5 Department of Primary Care, Azienda USLIRCCS of Reggio Emilia, 42122 Reggio Emilia, Italy; andreina.saba@ausl.re.it

**Keywords:** severe brain injury, palliative care, caregivers, neuro-palliative care, qualitative research, reflexive thematic analysis, dignity, quality of life, family-centred care

## Abstract

**Highlights:**

**What are the main findings?**

- Healthcare professionals identify complex and evolving palliative care needs in patients with severe brain injuries, strongly centred on dignity, quality of life, and communication challenges
- Caregivers experience significant emotional burden, anticipatory grief, and unmet support needs, which remain insufficiently addressed within current care pathways.

**What are the implications of the main findings?**

- Neuro-palliative care was perceived as highly relevant across the entire care trajectory of patients with severe brain injuries, not limited to end-of-life phases.
- The development of structured, multidisciplinary, and family-centred care models is essential to improve care quality and support both patients and caregivers.

**Abstract:**

**Background/Objectives**: Severe brain injuries generate complex, long-term needs requiring intensive physical, cognitive and relational care. These conditions also profoundly affect families, who often experience emotional distress, uncertainty and a heavy caregiving burden. Although neuro-palliative care is increasingly recognised, the early integration of palliative care for this population remains limited. This study aimed to explore healthcare professionals’ perceptions of the palliative care needs of patients with severe brain injuries and their caregivers and to identify factors that hinder or facilitate early palliative care implementation in specialised settings. **Methods**: An interpretive qualitative study was conducted using Reflexive Thematic Analysis. Fifteen semi-structured narrative interviews were carried out with healthcare professionals working in specialised hospital units in Northern Italy. Data were analysed inductively through an iterative and reflexive process following Braun and Clarke’s six phases. Methodological rigour and transparency were ensured using the COREQ checklist. **Results**: Five themes were identified: (1) intensive, individualised patient care needs with complex communication issues; (2) palliative needs centred on dignity, quality of life and early integrated management; (3) caregivers’ involvement and expectation-related difficulties; (4) continuous or anticipatory grief requiring structured psychological support; (5) facilitators and barriers influencing care pathways. **Conclusions**: Healthcare professionals identify intertwined and evolving palliative care needs in both patients with severe brain injuries and their families. The findings highlight the perceived importance of early, integrated and multidisciplinary neuro-palliative care models focused on dignity, symptom relief and sustained emotional support.

## 1. Introduction

Severe brain injuries, whether traumatic or non-traumatic, lead to a significant and prolonged disruption of normal brain function [1]. Their consequences are often profound and long-lasting, substantially affecting the quality of life of both patients and their families [2]. These conditions require major lifestyle adjustments, compelling individuals to establish a new sense of balance and reorganise their daily routines. This process generates specific needs, clinical, informational, communicative, emotional, psychological, and social, that must be adequately addressed [3]. As a result, care cannot be limited to physical treatment but must include continuous, person-centred support that encompasses emotional and relational dimensions [4].

A critical aspect of this process involves fostering acceptance and integration of a new personal identity, a challenge faced not only by patients but also by their families. Because adaptation is complex and influenced by multiple factors, tailored interventions are often required to integrate physical rehabilitation with psychological support, which remains frequently overlooked [5].

Despite this, many patients report unmet needs after hospital discharge, expressing feelings of abandonment, premature discharge, and inadequate post-acute support—factors that further compromise their quality of life [6].

Severe brain injuries also deeply affect family systems. The severity of the patient’s condition directly impacts caregivers’ psychological well-being, exposing them to intense emotional distress and substantial disruptions in their lifestyle [7]. Caregivers report evolving needs over time, largely related to medical information, professional support, involvement in care, and training for daily tasks [8]. Peer support, particularly contact with others who have shared similar experiences, is also identified as highly beneficial [9].

In addition to emotional strain and daily physical demands, financial difficulties are frequent and significantly contribute to caregiver burden [10]. Addressing these needs requires flexible, ongoing, and targeted interventions that provide financial, legal, managerial, and organisational support [11]. Many caregivers experience substantial stress, anxiety, and fatigue, highlighting the importance of offering regular respite, appropriate counselling, and supportive strategies throughout the caregiving trajectory [7].

Patients with severe brain injuries often experience a high symptom burden, exacerbated by cognitive and communication impairments that hinder clinical management and limit their ability to express needs [12]. As a result, essential needs frequently remain unmet due to inadequate care pathways and limited access to palliative services [13]. Although healthcare professionals acknowledge the relevance of palliative care in this population, its application in clinical practice remains limited and often restricted to end-of-life stages, revealing a gap between perceived utility and actual implementation [14].

The needs of patients with severe brain injuries are sometimes underestimated, resulting in reduced palliative support compared with other critically ill populations, despite evidence that early palliative care integration reduces unnecessary suffering, lowers healthcare costs, and improves overall quality of care [15]. When palliative care is integrated, benefits include more patient-centred decision-making, increased family involvement, and fewer aggressive interventions at the end of life [16]. Nonetheless, perceptual differences persist; clinicians often prioritise clinical and decisional aspects, whereas families more readily recognise the patient’s suffering and unmet needs, reflecting persistent discrepancies between clinical and family perspectives [12].

Healthcare professionals also acknowledge caregivers’ need for clear, timely, and understandable information regarding the patient’s health status, treatment, and the broader impact of the injury on daily life [17]. They emphasise the importance of open communication that does not avoid difficult news and the early involvement of families in the care process [18].

Caregivers frequently associate prognostic uncertainty with a need for hope. While nurses generally support and validate this need, physicians often express concerns that actively challenging or moderating such hope may undermine families’ coping abilities [19]. Consequently, caregivers’ needs—which begin at diagnosis and extend beyond the patient’s death—include support for both anticipatory and post-loss grief [20]. Caregivers experience profound sadness, fear, and emotional burden even before the actual loss, resulting in higher psychological stress compared with post-bereavement stages and requiring specific support interventions [21].

Despite awareness of these needs, professionals report limitations due to inadequate time, resources, and specialised training, which hinder their capacity to provide comprehensive family support. To address these challenges, healthcare workers highlight the importance of implementing dedicated resources and training programmes to enhance the recognition and management of families’ specific needs [22].

Given this context, the present study aims to explore the perceptions of healthcare professionals working in hospital settings within a Local Health Authority in Northern Italy regarding the palliative care needs of patients with severe brain injuries and those of their caregivers.

## 2. Materials and Methods

### 2.1. Study Design

This study adopted an interpretive qualitative design and used Reflexive Thematic Analysis (RTA) as its epistemological and methodological framework, following the approach proposed by Braun and Clarke [23,24]. Guided by an interpretivist and constructivist orientation, this study assumes that meaning is co-constructed through researcher–participant interaction and shaped by the social and contextual dynamics in which these experiences occur [25].

RTA was chosen to facilitate an in-depth exploration of how healthcare professionals construct meanings, articulate their positions, and describe their care practices within complex clinical contexts. This approach supports an active, engaged, and reflexive analytic process where the researcher plays a central interpretative role. Reflexive Thematic Analysis is well-suited to capturing implicit dynamics, emotional tensions and underlying value structures, offering the flexibility needed to examine diverse perspectives and sense-making processes [23,24]. Finally, the 32-item Consolidated Criteria for Reporting Qualitative Research (COREQ) checklist was utilised to ensure accurate, transparent, and standardised reporting (see Table S1 in Supplementary Materials) [26].

### 2.2. Participants and Setting

A convenience sample was recruited from healthcare professionals working in hospital units of a Local Health Authority in Northern Italy that provide care for patients with severe brain injuries. Members of the research team identified potential participants and contacted them through the nursing coordinators and the medical directors of the units. Those who agreed to participate were invited to in-person interviews held in hospital meeting rooms.

Potential participants were identified by the researchers based on the following inclusion criteria. Eligible participants were healthcare professionals, including physicians, nurses, physiotherapists, speech therapists, psychologists, and healthcare assistants, who were employed in the hospital units of the Local Health Authority and who provided care to patients with severe brain injuries. Healthcare professionals with less than one year of experience in this specific clinical field were excluded.

The research team provided detailed information about the study and interview procedures. Invited professionals received an information sheet and a consent form and were informed that the signed consent indicated a free and voluntary decision to participate. All contacted professionals agreed to take part in the interview.

The number of participants was determined by the depth and richness of the data rather than by saturation, in accordance with the principles of RTA. Data collection concluded when the material obtained was sufficiently rich to address the research questions [27].

During participant recruitment, principles of pluralism and heterogeneity were observed by including professionals with different roles, varying years of experience in the setting and an adequate representation of both men and women. The individuals invited to take part in the study were not in any position of dependence with respect to the researchers, and therefore their decision to provide consent was entirely free and made without any form of pressure.

### 2.3. Research Team

The research team consisted of nurses with expertise in qualitative research. All members of the nursing research team were women. AS (RN, MScN, PhDs) and LI (RN, MScN, PhD) were senior researchers with extensive experience in qualitative methodologies. FP, SC, LG (RN, MScN), and OD (RN, MScN, PhDs) contributed as research collaborators with training in qualitative inquiry. All members of the research team were employed at universities as researchers and adjunct faculty. None of the researchers had any prior relationship with the participants before the study.

### 2.4. Data Collection

A semi-structured narrative interview was used for data collection. This technique was considered suitable because the study aimed to explore complex or insufficiently understood phenomena, to gather data grounded in real-life situations and to answer open research questions that could not be limited by a structured questionnaire. The narrative interview also encouraged participants to express themselves freely, as the interviewer asked open questions, allowed ample time for responses and listened without judgement [28]. All interviews were conducted in person and were audio recorded with the participants’ consent. Healthcare professionals were asked to make themselves available for possible follow-up contact in case additional information was needed.

The interview questions were developed collectively through group discussions in order to ensure that they were open, non-directive, neutral, and conducive to reflection.

The interview guide was pilot tested with healthcare professionals to assess clarity and relevance; data from these pilot interviews were excluded from the final analysis.

The interview guide consisted of three sections, each containing at least one question designed to elicit information relevant to the aim of the study (Table 1).

During the in-person interviews, the interviewer ensured that the participants were in a suitable environment that guaranteed privacy, including a quiet space free from distractions, and created a climate of trust and attentive listening. This approach supported active engagement from the interviewees. Each interview was attended by an observer who took notes on the main elements of both verbal and non-verbal communication, generating field notes. The interviewers were two members of the research team (F.P. and S.C.); both were trained in qualitative interviewing, while the observers were the other two members (L.G. and O.D.).

Once the interviews were completed, they were assigned a numerical identification code to ensure anonymity, and a table was created containing basic biographical information for each participant.

### 2.5. Data Analysis

Data management involved audio-recording, verbatim transcription of interviews, anonymisation of transcripts, and organisation of data before analysis. Interpretive analysis was then conducted using reflexive thematic analysis, focusing on the identification of patterns of meaning and on an interpretive, reflexive process rather than purely descriptive coding.

Data analysis followed the six phases outlined by Braun and Clarke [24]. The analytic process began with familiarisation with the data through repeated reading of the transcripts and initial note-taking. This was followed by the generation of initial codes and the identification of preliminary meaning units, conducted independently by two researchers (F.P., S.C.). The third phase involved the initial search for themes by grouping meaning units into potential subthemes and themes. In the fourth phase, these analytic constructions were discussed within the research team to enhance reflexive depth. The fifth phase focused on defining, refining, and naming themes to ensure clarity and coherence. Finally, the sixth phase consisted of producing the analytic report. To ensure the scientific rigour of the analysis, the emerging themes were first reviewed independently and later discussed collectively within the research team [24,29].

All interviews were transcribed manually and verbatim in their entirety. The researchers conducted an inductive analysis and manually developed tree structures to move from meaning units to subthemes and central themes. All meaning units were then grouped, assessing whether some of the analytic constructions reflected shared conceptual patterns that could be integrated into broader common meanings. This process facilitated the identification of the subthemes and central themes. The emerging themes and subthemes were compared and discussed within the research group and were subsequently refined and renamed to improve their adherence to the data and to strengthen analytic coherence [30].

### 2.6. Rigour

To ensure the scientific rigour of the qualitative study, the research team referred to the criteria proposed by Morse. Rigour was pursued through attention to internal validity, reliability, and external validity, and through the continuous practice of reflexivity. Reflexivity was maintained throughout the entire analytical process, with all members of the research team adopting a reflective and critical attitude toward their interpretations.

Internal validity was supported by ensuring transparency in the analytic process and by providing a detailed description of the analytic phases and interpretative decisions. Reliability was addressed by documenting the data collection process in detail and by ensuring consistency in methodological procedures. External validity was considered in terms of the usefulness and relevance of the findings for other similar contexts, reflecting the transferability of the results rather than statistical generalisation [31].

## 3. Results

A total of fifteen interviews were conducted with healthcare professionals, including nurses, physicians, healthcare assistants and psychologists, between December 2025 and January 2026. Nine participants were women, and six were men, with a mean age of forty-five years (range: thirty-two to fifty-eight years). Seven participants were nurses, five were healthcare assistants, two were physicians, and one was a psychologist. The interviews lasted an average of thirty-five minutes. All participants worked in the progressive care centre of a hospital in Northern Italy, with an average length of service of six years (range: one to twenty years).

The analysis of the interviews generated a total of 246 meaning units. Five main themes emerged: (i) needs of patients; (ii) palliative care needs of patients; (iii) needs of caregivers; (iv) palliative care needs of caregivers; and (v) facilitators and barriers. All themes reflect healthcare professionals’ perceptions and interpretations of the needs of patients with severe brain injury and their caregivers. Themes, related subthemes, and examples of codes are presented in Table 2 and Table 3.

### 3.1. Theme 1: Patients’ Needs

Within Theme 1, “Patients’ Needs,” healthcare professionals described two subthemes that emerged: Subtheme 1.1. “Specific Caregiving Needs and Humanisation of Care” and Subtheme 1.2. “Need Related to Communication Difficulties.”

In the first subtheme, “Specific Caregiving Needs and Humanisation of Care,” patients’ needs were described by professionals as primarily related to intensive, continuous and highly personalised care that involves both the patient and their family. Care was often defined as a *“comprehensive caregiving process for the whole family” (cod 1.1)*, highlighting that the need for care cannot be limited to the patient alone but requires attention to the broader relational context.

From an operational perspective, primary care needs emerged strongly. These included mobilisation, hygiene, nutrition, bowel management and the daily monitoring of vital parameters. Participants described a structured care routine, aimed especially at preventing complications such as skin lesions and infections, in patients portrayed as *“very fragile” (cod 8.1).* The use of specific equipment, such as pressure-relieving mattresses, appropriate hygiene aids and frequent repositioning, was considered a *“central element in the quality of care” (cod 10.1).*

Alongside this technical and assistive dimension, professionals underscored the importance of maintaining close attention to *“the patient’s residual resources” (cod 11.12)*, even in conditions of total dependence. They described the need to observe minimal bodily or behavioural cues to detect possible pain or discomfort, recognising what *“remains” (cod 11.2)* of the patient’s expressive abilities.

Humanisation of care also emerged through the attempt to recreate an environment that feels as familiar and meaningful as possible, through the presence of personal objects, photographs or items that recall the patient’s previous life. The possibility of *“having their own blanket” (cod 4.1)* or images from their home environment was described to preserve identity continuity and enhance a sense of safety and recognition.

In the second subtheme, “Need Related to Communication Difficulties,” professionals described communication as one of the most complex and challenging areas of care. These difficulties arose both in interactions with the patient and in communication with the family, especially in the presence of severe motor and cognitive impairments.

Regarding the patient, communication was often limited or non-verbal, making it difficult to understand their real needs. Professionals reported having to *“interpret minimal expressive signals, such as eye gaze”* or facial expressions when trying to understand what the patient *“wants to communicate” (cod 15.3)*.

A recurring issue was the challenge of distinguishing physical pain from psychological or relational discomfort. Participants described how states of apparent severe distress could be linked to simple needs, such as the *“desire to be mobilised, cared for or simply to have company, for example by having the television on” (cod 10.3)*.

At the same time, participants highlighted communication challenges with families, particularly regarding the management of expectations. In cases of severe injuries and high levels of dependence, families may hold expectations of recovery that are not consistent with the actual clinical condition, *“leading to misunderstandings and tension within the care relationship” (cod 9.2).*

### 3.2. Theme 2: Patients’ Palliative Care Needs

Within Theme 2, “Patients’ Palliative Care Needs,” healthcare professionals described two subthemes that emerged: Subtheme 2.1. “Need for Dignity and Quality of Life” and Subtheme 2.2. “Need for Early and Integrated Palliative Care.”

Within the subtheme “Need for Dignity and Quality of Life”, participants described dignity and quality of life as closely interconnected dimensions of care. The need for dignity emerged as particularly relevant and was described by professionals as a primary objective of care. Quality of life was presented as a guiding criterion for clinical and assistive interventions, shaping decision-making and how patients are supported. In this regard, professionals emphasised how essential it is to *“always carry out an assessment aimed at improving quality of life” (cod. 12.5).*

Quality of life assessment was described as a continuous and dynamic process that considers both the clinical progress of the patient and their subjective experience of illness. This assessment is not delegated to a single practitioner but is constructed collaboratively, with team discussions enabling the identification of emerging needs and the monitoring of changes in the patient’s condition. As participants reported:

*“We carry out an assessment as a team, and from there we identify the needs, the quality of life of the patient, whether the condition is deteriorating or not” (cod. 13.11).*

A key aspect related to the dignity of the person concerns the early management of symptoms, considered essential for reducing both physical and emotional suffering. Professionals highlighted the importance of actively supporting patients in recognising and reporting symptoms from their earliest onset, through encouragement and ongoing assistance. They underscored the value of *“prompting them to report symptoms early,”* even when these involve initial signals such as *“a slight pain or shortness of breath” (cod. 14.13).*

Overall, the need for dignity and quality of life emerged as a multidimensional construct that integrates clinical assessment, team-based decision-making, attention to the patient’s subjectivity and early symptom management. This perspective reflects a model of palliative care oriented not only toward the disease but toward the person.

**The second subtheme** concerned the need to activate early and integrated palliative care, considered an essential component of the care trajectory for patients with severe brain injuries. The decision to initiate such care was described as the outcome of a shared team evaluation that included the use of pain scales, observation of clinical parameters and general condition, and ongoing involvement of caregivers. As participants noted:

*“We always evaluate as a team… always involving caregivers and sharing with them the need to eventually transition to a palliative care approach” (cod. 10.8).*

Some participants expressed the idea that patients with severe brain injuries inherently fall within a palliative care pathway, regardless of the stage of illness. From this perspective, it was stated that *“all patients with severe brain injuries… enter by default into a palliative care pathway” (cod. 9.8)* and that *“they should all enter a palliative care regimen” (cod. 3.8).* These statements reflect the perspectives of some participants and should not be interpreted as a uniform or universal clinical indication for all patients with severe brain injury.

Finally, the activation of palliative care was described as a multidisciplinary process that considers the patient’s wishes and involves various professionals, including neurologists and palliative care specialists. This pathway was portrayed as gradual and shared:

*“There is the entire multidisciplinary pathway… there is the will of the patient… the multidisciplinary team meets… and it is mainly the neurologist who decides when it is time to activate the pathway” (cod. 14.6).*

### 3.3. Theme 3: Caregivers’ Needs

Within Theme 3, “Caregivers’ Needs,” healthcare professionals reported that two subthemes emerged: Subtheme 3.1. “Need for Caregiving Support for Their Loved One” and Subtheme 3.2 “Managing Expectations and Non-Acceptance.”

In the first subtheme, “Need for Caregiving Support for Their Loved One,” caregivers expressed a strong desire to continue feeling actively involved in the care of their relative and to maintain a meaningful role within the assistive pathway. This need was described as the wish to *“continue being a resource for their family member” (cod. 2.17)*, even when the patient’s clinical condition was severely compromised. However, such involvement could sometimes translate into forms of over-care, characterised by excessive attention and care behaviours perceived as intrusive or clinically inappropriate. Some participants observed:

*“An excessive level of attention… even too much for this type of patient, which leads family members to intervene directly with care procedures, such as suctioning, which are not always appropriate” (cod. 10.5).*

These behaviours were interpreted as expressions of profound emotional ambivalence. On the one hand, there is a strong impulse to care or overcare, often linked to feelings of guilt; on the other, there may be the simultaneous and conflicting desire to *“let them go” (cod. 9.6)*. This internal tension can coexist within the same individual, reflecting the emotional complexity of caregiving in the context of severe brain injury.

In the second subtheme, “Managing Expectations and Non-Acceptance,” caregivers were described as needing support in managing expectations and accepting the illness, which participants identified as one of the most challenging aspects of care. Professionals reported being able to recognise early signs of *“exaggerated apprehension,” “insecurity”* or *“complete non-acceptance” (cod. 10.4)*, which guide the multidisciplinary team in initiating timely supportive interventions, often involving psychologists and physicians.

A central element is the need to help caregivers recalibrate their hopes, supporting them in navigating a difficult balance between caring for their loved one and acknowledging the limits imposed by the clinical condition. This process was described as particularly demanding, as it requires holding together the desire to care with the awareness that *“I cannot hold on too much hope”* or that it is necessary to *“reframe expectations” (cod. 2.9)*. This difficulty affects not only caregivers but also the professionals providing care.

Acceptance of the illness was described as one of the most significant challenges faced by families and was referred to as *“truly a tragedy”* and *“the greatest difficulty”* observed in daily practice (cod. 10.4). Failure to accept the condition of their loved one can generate relational tension and, in some cases, *“a verbal confrontation”* between family members and staff (cod. 4.7).

Caregivers’ expectations were often oriented towards possible clinical improvements, such as “*awakenings*,” functional recovery, or the restoration of verbal communication, thereby widening the gap between clinical reality and family members’ emotional experience (cod. 13.4). This highlights the need for continuous accompaniment that supports not only the transmission of information but also the emotional processing of the illness and its outcomes.

### 3.4. Theme 4: Caregivers’ Palliative Care Needs

Within Theme 4, “Caregivers’ Palliative Care Needs,” according to healthcare professionals, two subthemes emerged: Subtheme 4.1. “Continuous Grief” and Subtheme 4.2. “Need for Psychological Support.”

In the first subtheme, “Continuous Grief,” caregivers were described as needing support in dealing with an ongoing form of grief linked to the existential suspension in which their loved one remains. Family members were often perceived as unable to fully acknowledge the irreversibility of the situation, living in the hope of a possible awakening despite being aware that the patient is in *“a limbo from which they cannot emerge” (cod. 1.4)*. This condition was interpreted as a form of grief that cannot be processed, one that persists over time and requires specific psychological and emotional support. Participants emphasised that family members *“need to be supported because for them it is a continuous grief” (cod. 1.12),* highlighting the importance of sustained emotional accompaniment.

The grief experienced by caregivers was also described as potentially pathological, since the clinical condition of the patient prevents the activation of a traditional grieving process. As noted by participants, *“the grief is never processed or elaborated by the family members” (cod. 8.8).* This perspective underscores the necessity of palliative care interventions aimed not only at the patient but also at caregivers who are navigating an unresolved and unresolvable loss.

The second subtheme, “Need for Psychological Support,” concerns caregivers’ need for structured and continuous psychological support, considered a central component of palliative care addressed to the family system. Professionals noted that caregivers *“really need to have meetings with the psychologist” (cod. 10.6)*, and in some cases, even this level of support was perceived as insufficient. Caregivers’ psychological needs were described as a priority and, for some participants, as *“the most important” (cod. 5.5)* within the care pathway.

Specialised psychological support was considered necessary not only during the patient’s stay in the facility but also from the earliest stages of the illness trajectory. Participants highlighted the absence of psychological accompaniment before admission and the need for such support to be available *“from the moment of the injury through to the end of the pathway” (cod. 9.7)* to help families understand the situation and adjust to their new reality.

Psychological support was also recognised as a service actively sought by caregivers. Professionals reported that *“many of them specifically ask for it” (cod. 13.14)* once informed of the availability of psychological services. Alongside professional support, caregivers valued an empathic and reassuring presence from the healthcare team, including *“a word of comfort” (cod. 14.16)* and gestures of closeness aimed at conveying the team’s commitment to ensuring that their loved one receives the best possible care, with attention to reducing suffering and pain.

### 3.5. Theme 5: Facilitators and Barriers

Within Theme 5, “Facilitators and Barriers,” healthcare professionals described two subthemes that emerged: Subtheme 5.1. “Facilitators” and Subtheme 5.2. “Barriers.”

In the first subtheme, “Facilitators,” several factors were identified as supporting the care pathway of both patients and caregivers. A key facilitator was the in-depth knowledge of the patient, which enabled professionals to better interpret their needs:

*“The better we know the patient, the more we can understand and interpret their needs” (cod. 10.12).*

Another facilitating factor concerned the admission phase, described as a crucial moment in the care process. Participants stressed the importance, from the patient’s first arrival of ensuring *“abundant communication and being extremely clear” (cod. 10.21)*, aspects considered fundamental for shaping a shared pathway over time.

Participants also highlighted the value of consistency and coherence in communication across the various services involved in the care process. The need to *“provide the same responses” (cod. 7.12)* across different care settings, from acute hospital wards to rehabilitation units, emerged as a key factor in reducing confusion and disorientation among family members. Finally, participants recognised multidisciplinary assessment as an additional facilitator, underscoring that decisions are not made *“independently by a single professional” (cod. 4.11)* but rather through collaborative teamwork.

In the second subtheme, “Barriers,” several obstacles emerged that may complicate the care of patients and caregivers throughout the assistive pathway. One barrier concerned the physical environment and the structural setting, which in some cases was perceived as inadequate or uncomfortable. Participants noted that factors such as excessive light, non-adjustable curtains, stains on the walls or humidity issues could affect patient well-being and compromise the quality of the admission experience:

*“Even the setting of the facility plays a role… at times there was dissatisfaction… too much sunlight shining on the patient’s face… patients must be welcomed completely” (cod. 10.23).*

Another barrier was the lack of clarity and coherence in communication within the care pathway. Participants noted that inconsistent responses from staff could generate confusion and conflict, especially among caregivers who did not clearly understand the sequence of procedures:

*“If we give completely different answers, we will have people who do not know where to go and have no idea what will happen next, and conflicts arise as a result” (cod. 9.12).*

Administrative procedures were also identified as a limiting factor, as they can slow access to interventions that improve patient comfort, such as palliative care. Participants highlighted that although faster action would be possible, *“bureaucracy extends the timeframe” (cod. 15.7).*

Finally, a barrier related to staff trust emerged. Distrust from some patients or caregivers could generate negative perceptions and unusual thoughts, creating tension in the relationship with the facility. As one participant stated:

*“There was always someone who, being distrustful of us, spread unusual thoughts about everyone” (cod. 13.7).*

## 4. Discussion

This study explored healthcare professionals’ perceptions of the palliative care needs of patients with severe brain injuries and their caregivers, providing insight into how these needs are understood and interpreted in specialised care settings. The first theme, patients’ needs, revealed the requirement for intensive, continuous and highly personalised care, which extends beyond the management of primary care needs. The second theme, patients’ palliative care needs, highlighted the centrality of dignity and quality of life as guiding principles of care, as well as the importance of early symptom management and shared multidisciplinary assessment. The third theme, caregivers’ needs, showed their strong desire to maintain an active role in their loved one’s care, sometimes expressed through forms of overcare, and their difficulty in managing expectations concerning possible clinical recovery. The fourth theme addressed caregivers’ palliative care needs, characterised by experiences of continuous or anticipatory grief and the need for structured and ongoing psychological support throughout the illness trajectory. Finally, several facilitators emerged, including coherent team communication, multidisciplinary collaboration and in-depth knowledge of the patient. In contrast, structural and organisational barriers such as inconsistent information, interprofessional difficulties and bureaucratic constraints were identified as factors that may hinder early palliative care integration and contribute to conflict with families.

Although several themes identified in this study align with existing neuro-palliative care research, this convergence may reflect both the robustness of prior evidence and the influence of shared professional discourses within specialised care settings. In line with a reflexive thematic analysis approach, themes were interpreted as analytical constructions, co-constructed through participants’ accounts and researchers’ analytic engagement rather than as purely inductive or theory-free categories.

A central issue emerging from this study, according to healthcare professionals, concerns the substantial difference between oncology patients and those with chronic severe brain injury in the context of palliative care. In oncology, disease trajectories are generally more predictable, and palliative care integration is now widely recognised and embedded across the entire continuum of care [32]. Conversely, in patients with severe acquired brain injury, the clinical course is marked by prognostic uncertainty, fluctuations and complex chronicity, making it more difficult to identify the appropriate moment for palliative care activation. As highlighted in the neuro-palliative care literature, palliative care should be integrated early for patients with severe neurological conditions, not only in the terminal phase but throughout the illness trajectory, with a focus on quality of life and symptom management [1,13].

A prominent finding concerns healthcare professionals’ perceptions of caregivers’ non-acceptance of the patient’s clinical condition and the challenges linked to managing their expectations. These aspects were closely related to the subthemes of overcare and difficulty reframing hope. Caregivers’ expectations were often oriented toward unrealistic possibilities of improvement, particularly regarding the recovery of communication or a return to premorbid functioning, sometimes due to a partial understanding of diagnosis and prognosis. Consistent with the literature, caregivers’ expectations tend to be more optimistic than those of healthcare professionals, which may generate tension and conflict [16,18]. In this context, hope plays an ambivalent role: on the one hand, it functions as a necessary coping strategy; on the other, it may hinder acceptance of clinical reality. Participants therefore stressed the importance of reframing hope through clear, honest and empathic communication that maintains meaning without fostering unrealistic expectations [19]. This reflects a genuinely family-centred care model in which caregivers are recognised as integral to the care pathway and as recipients of dedicated interventions [33].

Another key aspect concerns dignity and quality of life, which emerged as guiding criteria of care. Dignity was reflected in attention to personal identity, the care environment, individualised interventions and recognition of minimal signs of suffering, extending beyond physical symptoms to include emotional, social and existential dimensions [1]. Quality of life assessment was described as a continuous and dynamic process, closely linked to the patient’s clinical trajectory and subjective experience of illness. Within this framework, symptom control emerged as a primary need, consistent with existing research [6,34].

Another important finding concerns the care environment and the structural setting as integral components of dignity. The structural barriers described in the results, such as inadequate physical spaces, limited comfort and bureaucratic delays, demonstrate that quality of life does not depend solely on clinical intervention but also on organisational and environmental factors, reinforcing the notion of holistic care [35].

In the effort to enhance person-centred care, the need for early and integrated palliative care emerged strongly. However, palliative care continues to be associated with critical decision points or end-of-life stages rather than being seen as an inherent component of the care trajectory, consistent with previous literature. The aim of palliative care is instead to ensure the best possible quality of life regardless of disease stage, through patient-centred care, shared decision-making and family involvement. According to participants, early integration of palliative care may improve quality of care, reduce disproportionate interventions and foster greater understanding of the patient’s condition among family members [15]. This qualitative study does not aim to define clinical referral criteria, triggers, or timing thresholds for neuro-palliative care. Future research should identify and validate operational indicators to guide timely integration across heterogeneous severe brain injury trajectories.

In this context, these findings also raise relevant ethical and communicative issues that are inherent to the care of patients with severe brain injuries. Decision-making capacity, prognostic uncertainty, and the communication of potentially life-limiting trajectories represent complex challenges for both clinicians and families, especially in relation to decisions about withholding or withdrawing life-sustaining treatments. Although these aspects were not the primary focus of this study, they emerge as critical areas requiring further exploration within neuro-palliative care practice and research.

Finally, anticipatory grief emerged as a significant theme, closely linked to the concept of “ambiguous loss,” in which the patient is physically present but cognitively and relationally altered. This existential suspension places caregivers in a state of ongoing, unresolvable grief that exposes them to significant psychological distress. The literature describes bereavement support as a preventive palliative care intervention aimed at families, in which accurate information and early identification of vulnerable caregivers may reduce the risk of complicated grief and improve both care quality and caregivers’ emotional well-being [20,36].

### Strengths, Limitations and Implications

From the perspective of healthcare professionals, the findings of this study contribute to understanding how the needs of patients with severe brain injuries and their caregivers are perceived in specialised care settings. The interviews were organised and conducted with careful attention to ensure a consistent approach across all participants, which strengthens the credibility of the results. However, the use of convenience sampling and recruitment through internal coordinators may have introduced selection (gatekeeper) bias, potentially favouring the participation of professionals who were more engaged or aligned with institutional perspectives. Moreover, only some professional roles within the selected care settings were included, and therefore, the perspectives of all healthcare professionals involved in the care of these patients and their families, such as physiotherapists and speech therapists, were not captured. Furthermore, this study focused exclusively on the perspectives of healthcare professionals, leading to an interpretative reading of the needs of patients with severe brain injuries and their caregivers. The absence of direct input from patients and caregivers also limits analytical triangulation and requires cautious interpretation of findings related to their needs. In addition, some thematic areas are conceptually adjacent and partially overlapping (e.g., caregivers’ needs and caregivers’ palliative care needs), reflecting the intertwined nature of needs in this context and representing an analytic limitation.

This study was conducted in two specialised facilities dedicated to the care of this patient population, which may limit the applicability of the findings to other care contexts. This context specificity may influence the transferability of the findings to other healthcare settings with different organisational and professional characteristics. In addition, the study was conducted within a single Local Health Authority, which may limit transferability to regions or organisations with different service structures and care pathways. It is therefore necessary to assess how professionals’ perceptions and the needs of patients and caregivers may vary across different settings, considering the organisational and cultural characteristics of different healthcare structures. In light of this, future research should involve diverse care environments, include a larger and more heterogeneous group of healthcare professionals, and integrate the perspectives of patients and family members to better understand their actual needs and to further advance knowledge in this field. Additionally, a few explicitly divergent or contradictory perspectives emerged within the dataset, which limits negative case analysis and interpretative balance.

The results provide valuable insights for clinical practice, professional training and the development of tailored interventions aimed at improving the quality of care for patients with severe brain injuries and the support offered to their caregivers. These findings represent an important starting point for the design of care models that incorporate the early and integrated implementation of palliative care for both patients and families. The effectiveness of such approaches should be further explored and evaluated in future studies.

## 5. Conclusions

Based on healthcare professionals’ perspectives, this qualitative study highlights perceived palliative care needs of patients with severe brain injury and their caregivers. In conclusion, this study confirms the high level of care complexity associated with patients with severe brain injuries and the significant impact of this condition on their families. According to healthcare professionals, the findings highlight that the early and systematic integration of palliative care is perceived as a highly relevant strategy for improving the quality of care, making it more humane, personalised, and person-centred.

This study also opens the way for future developments, such as further exploration of strategies to integrate palliative care within neurological settings and the investigation of patients’ and caregivers’ own perceptions of such care. These lines of inquiry may help bridge the gap between theory and practice and foster an integrated care model oriented toward quality of life.

## Figures and Tables

**Table 1:** Main interview topics and example questions.

Topics	Example Questions
Needs of Patients	Based on your clinical experience, could you describe the principal needs and challenges encountered by patients living with severe acquired brain injury?
Needs of Caregivers	Drawing on your professional experience, could you outline the main needs and difficulties faced by the caregivers of individuals with severe acquired brain injury
Palliative Care Needs	From your clinical perspective, how do you assess the potential palliative care needs of patients with severe acquired brain injury?

**Table 2:** Themes 1 and 2, related subthemes and examples of significant excerpts.

Themes	Subthemes	Examples of Significant Excerpts
1. Patients’ Needs	1.1 Specific Caregiving Needs and Humanisation of Care	*“Having with them a personal object, some photographs that bring them back a little to their own environment” (4.1)* *“Maintaining humanity and respect for the person, beyond the pathology” (7.20)*
	1.2 Need Related to Communication Difficulties	*“The main problems are communicative issues with the family.” (9.1)* *“Their problem is communication (…) but sometimes you can clearly see that they want to communicate something, and it becomes difficult to understand what they are trying to express.” (15.1)*
2. Patients’ Palliative Care Needs	2.1 Need for Dignity and Quality of Life	*“I find myself more in difficulty when dealing with needs related to providing quality of life.” (2.4)*
	2.2 Need for Early and Integrated Palliative Care	*“Starting from the assumption that, for me, they all need palliative care.” (1.5)* *“I believe that all patients with severe brain injuries should enter a palliative care pathway.” (9.8)*

**Table 3:** Themes 3, 4 and 5, related subthemes and examples of significant excerpts.

Themes	Subthemes	Examples of Significant Excerpts
3. Caregivers’ Needs	3.1 Need for Caregiving Support for Their Loved One	*“We have family members who are extremely demanding and who do not realise the extent of the patient’s severe loss of major brain functions, and they do not acknowledge it.” (7.13)* *“Having a living person, a living body with a mind or a thought or a consciousness that is no longer present creates, on the one hand, in family members the desire to care for or over care out of guilt, and on the other hand the wish to let them go.” (9.6)*
	3.2 Managing Expectations and Non-Acceptance	*“They expect awakenings, they expect improvements, they expect their loved ones to stand up again, to be able to speak.” (13.4)* *“A family member certainly does not* *accept the illness of their son, daughter or husband… and many times this can lead to a verbal confrontation.” (4.7)*
4. Caregivers’ Palliative Care Needs	4.1 Continuous Grief	*“From an emotional point of view, one experiences a pathological form of grief with these patients, because they remain suspended between life and death, and therefore the grief is never fully processed.” (8.2)*
	4.2 Need for Psychological Support	*“The psychological needs of caregivers are, in my opinion, the most important.” (6.16)* *“Even a small amount of external support would be greatly appreciated.” (15.11)*
5. Facilitators and Barriers	5.1 Facilitators	*“The main facilitator is for everyone to move in the same direction. If the physician says one thing and the nurse says another, this creates confusion for the family. Consistency and the ability to work in harmony are essential.” (7.22)* *“We should provide the same responses across all hospital facilities that are rehabilitative.” (9.11)*
	5.2 Barriers	*“The barriers are the difficulty in communication between different teams and the differing care paradigms.” (7.23)* *“For us, it feels like we are constantly struggling more with the family members than with the patient.” (13.6)*

## Data Availability

Due to privacy and ethical restrictions, the datasets generated and analysed during the study are not publicly available. However, anonymised data may be made available from the corresponding author upon reasonable request.

## Supplementary Materials

The following supporting information can be downloaded at: https://www.mdpi.com/article/10.3390/brainsci16050482/s1, Table S1: COREQ Checklist.

## Abbreviations

The following abbreviations are used in this manuscript:

AVEN	Area Vasta Emilia Nord (Northern Emilia Wide Area)
COREQ	Consolidated Criteria for Reporting Qualitative Research
RTA	Reflexive Thematic Analysis
